# From Control to Clue: Integrating Olfaction into the Object-Choice Task for Domestic Dogs (*Canis familiaris*)

**DOI:** 10.3390/ani16091324

**Published:** 2026-04-26

**Authors:** Sylvie Bergquist, Alexandra Horowitz

**Affiliations:** Dog Cognition Lab, Department of Psychology, Barnard College, New York, NY 10027, USA; ahorowit@barnard.edu

**Keywords:** canine olfaction, dog cognition, object-choice task, olfactory point, odor trail, decision-making

## Abstract

Humans rely heavily on vision for information-gathering, decision-making, and communication, making it difficult to imagine how our perception of the world might change if our primary sensory modality were entirely different. Dogs (*Canis familiaris*), for instance, rely as much or more on olfaction as on vision in information-gathering. Nonetheless, canine cognition research has largely emphasized visual tasks. In the present study (N = 48 dogs) we aim to begin to remedy this by designing an olfactory version of a prototypical dog-cognition experimental design: the object choice test. In the standard design, subjects respond to an experimenter’s pointing gesture to choose between two overturned cups, one of which is baited with a food treat. We extended this paradigm by adding trials using an “olfactory point” in place of the visual gesture. In these trials, two cotton strings extended from the cups toward the subject and converged in front of the subject. The string leading to the baited cup was scented with either the odor of the treat or the subject’s owner, while the string leading to the non-baited cup remained unscented as a control. Subjects followed both visual and olfactory points at rates significantly above chance. These findings suggest that dogs can use experimentally presented olfactory cues to guide choice behavior, supporting the development of experimental designs that better reflect species-specific sensory systems.

## Simple Summary

Dogs experience the world very differently from humans, relying heavily on their sense of smell rather than vision alone. However, most studies of dog cognition and behavior have focused on what dogs can see, not what they can smell. This study set out to address that gap by testing whether dogs could use odors in a structured way to guide decision-making, as they use visual cues. We adapted a common task in dog research, in which dogs choose between two containers based on a human pointing gesture, by creating a scent-based “point.” In lieu of a hand gesture, dogs were given a scent trail leading to the correct container, using either the smell of a treat or the smell of their owner. We found that dogs were able to follow both visual and scent-based cues at rates higher than chance, indicating that dogs successfully use smell to make choices. This shows that scent can act as a meaningful signal for dogs, not just a background factor. These findings are important because they encourage scientists to design studies that better match how dogs naturally gather information.

## 1. Introduction

Canine cognition work has a smelling problem. Most research involves visual cues and visual information, though the subjects, domestic dogs (*Canis familiaris*), are predominantly olfactory. Many experimental paradigms have been taken from paradigms used with humans and nonhuman primates and focus on problem-solving in a visual domain. For the most part, possible odor signals in the experimental environment are rarely incorporated into the experimental design or controlled in a systematic way. This omission may lead researchers to misinterpret subject behavior, as cues available to the dog through olfaction may go undetected by experimenters operating within a visually oriented framework [1]. If cognition research aims to understand how animals solve problems, then experimental designs must account for the sensory modalities through which those animals naturally acquire information. 

In a review of 481 canine cognition studies from 2008 to 2018, only 56 explicitly mentioned odor controls in their methods. Even these were more often food odors; very few were biological odors [1]. This is surprising, given the widespread knowledge about the performance of working dogs in finding people and detecting chemical and biological odors by scent alone [2,3]. Recently, a few research programs within comparative cognition have centered around olfaction and found that dogs can discriminate emotional odors of humans [4], other dogs [5], represent toys [6], and people [7] by odor, and potentially represent themselves [8]. These findings make clear that, within this line of research, olfaction is not simply a nuisance variable that needs to be controlled for but is instead a potential source of information for the dog. By trying to incorporate this sensory modality more fully into cognition research, researchers may better understand factors which drive canine decision-making and problem-solving.

One of the most repeated tasks in canine cognition research, seen as fundamental to understanding cognition, is the object-choice task (OCT) [9]. In this paradigm, subjects must use the gestural communication of a human experimenter (such as gaze or pointing) to guide their choice between two objects—typically overturned cups or buckets—in search of a hidden treat. Dogs have been shown to reliably follow human cues to solve the task [10,11,12], often performing at higher rates than apes or chimpanzees despite the latter species’ closer genetic similarity to humans [13,14]. However, these differences may be influenced by methodological factors and variation in social experience or rearing conditions, as dogs with limited human interaction (e.g., kennel-housed populations) often show reduced performance on the task [15,16]. Dogs are particularly responsive to pointing gestures when they are preceded by ostensive communicative signals such as eye contact, vocalizations, or other attention-calling behaviors [17,18,19,20]. Despite the extensive literature demonstrating dogs’ sensitivity to human gestures, researchers continue to debate how dogs interpret these cues. Some argue that dogs treat pointing as a communicative signal conveying information about a location, while others suggest that dogs may instead learn to associate human gestures with reward outcomes through repeated interactions [21,22].

The use of gazing and pointing cues emerges from the comparative nature of the task and their use in human and nonhuman primate research. Odor cues are often thought to be “controlled” or are ignored in experimental design [1,23]. As a result, the OCT reflects a human-centered sensory framework that does not fully align with the dog’s perceptual experience of the world [1]. It is an open question whether equivalent information presented in a more dog-appropriate modality would support similar task performance.

At a certain point, researchers began incorporating odor controls into object-choice tasks to address concerns that dogs might simply smell the baited container. These controls typically involved rubbing odor beneath both containers, baiting both containers, or baiting neither container [22,24,25,26]. Since then, this approach has largely remained standard practice, with odor treated primarily as a potential confound to be controlled rather than as a modality that could be explored further. Recent large-scale work from the ManyDogs Project has replicated dogs’ pointing comprehension across laboratories, and also relied on separate “smell-only” non-gestural control trials rather than integrating olfaction directly into the pointing trials themselves [22,27,28]. In these trials, one container was baited, but no pointing or other communicative cue was provided, ostensively requiring subjects to rely on olfactory information alone.

Despite dogs’ relatively weak performance in odor-control trials of the OCT, it is possible that their behavior reflects not an inability to smell but the task demands of the experimental design. Dogs experience up to 16 trials with pointing gestures before they are exposed to control trials [22], and it cannot be assumed that a dog will continue to understand the task once all human gestural components have been removed. This point is especially salient given that most subjects are owned by domestic dogs, which are highly familiarized with waiting for a human to provide some form of directional or communicative cue before acting.

The present study represents a complementary approach to the OCT: designing and enacting an olfactory analog to the pointing gesture. The goal of the present study was therefore not to propose a better olfactory control for the object-choice task. Rather, we aimed to investigate what subject dogs could accomplish with olfaction within the structure of an object-choice paradigm. To do this, we introduce an “olfactory point,” designed as a complement to the standard visual pointing cue. In doing so, we aim to explore the capabilities of the dog *qua* dog, rather than treating the dog solely as another comparative psychological subject. By using olfactory gestures, we seek to contribute to the understanding of how dogs use odor cues and whether odor can function communicatively to guide problem-solving even within a human–dog interaction context.

The present study consists of two parts: first, a standard object-choice task using visual pointing, modeled after the ManyDogs Project [22] protocol. The second part employs the same object-choice structure but replaces the visual point with an olfactory point: a cotton string extending from the cups toward the subject and carrying an odor cue and thus creating a scent trail toward the baited cup [29,30]. Two kinds of odorants were used for each subject: the scent of a treat or the scent of a familiar person (the dog’s owner) [31]. A control string with no added odorant was used for the non-baited cup. Throughout, we use the term “olfactory point” to refer to this communicative cue in a functional sense—as directing the subject’s attention toward a location—while “scent-trail” refers to its physical implementation.

We aimed to determine whether pet dogs could use this odor “point” in a manner analogous to their use of pointing and gaze cues to solve the task. If dogs successfully use the olfactory point, this supports the thesis that odors in testing environments may function as meaningful information rather than merely nuisance variables to be controlled. Such findings would further encourage the development of experimental paradigms that more closely reflect a canid-centered sensory experience.

## 2. Materials and Methods

### 2.1. Subjects

Dogs and owners were recruited through the Barnard Dog Cognition Lab database. Forty-nine dogs (21 M and 28 F) and their people were enrolled in this study. One subject was excluded due to being too nervous to participate in trials, resulting in a final sample of forty-eight dogs. Subjects were pre-screened to ensure that they were at least 6 months old, fully vaccinated, healthy, and comfortable interacting with new places and new people. The mean age was 5 years 5 months (range: 1 year–13 years 7 months). All but 4 dogs were spayed or neutered. Nineteen dogs were reported by their owners to be purebred, and twenty-nine were reported as mixed breed (Table 1; Phases A and B are described in detail in the Experimental Design section below).

### 2.2. Experimental Design

Prior to participation, owners completed an online survey asking questions about their dog’s age, breed (if known), weight, history of scent work, level of food motivation, eagerness to smell, and fear of new places. Food motivation, eagerness to smell, and fearfulness were rated on 4-point Likert scales (0–4). Dogs scoring below 3 on food motivation or eagerness to smell, or above 2 on fearfulness, were excluded from the study to ensure sufficient engagement with the task and comfort in the testing room. Experimental trials were run from February to May 2025 (Phase A) and October to December 2025 (Phase B). The overall study design and procedures were consistent across testing periods, with minor adjustments as noted below.

Subjects and owners came to the Dog Cognition Lab on Barnard College’s campus in New York City for a single lab visit. Consent was obtained from dog owners either electronically prior to the visit or in person prior to any data collection. Owners also received written instructions outlining their role during the study. Each visit lasted approximately 20 min and was conducted on weekdays between 5:30 p.m. and 7:30 p.m. Appointments were scheduled at least 30 min apart to prevent interactions between participating dog-owner dyads. Owner-subject dyads remained together for the entirety of the visit, and subjects had ad lib access to water. Upon completion, subject dogs received a graduation certificate and a toy.

### 2.3. Testing Room

**Figure 1:**
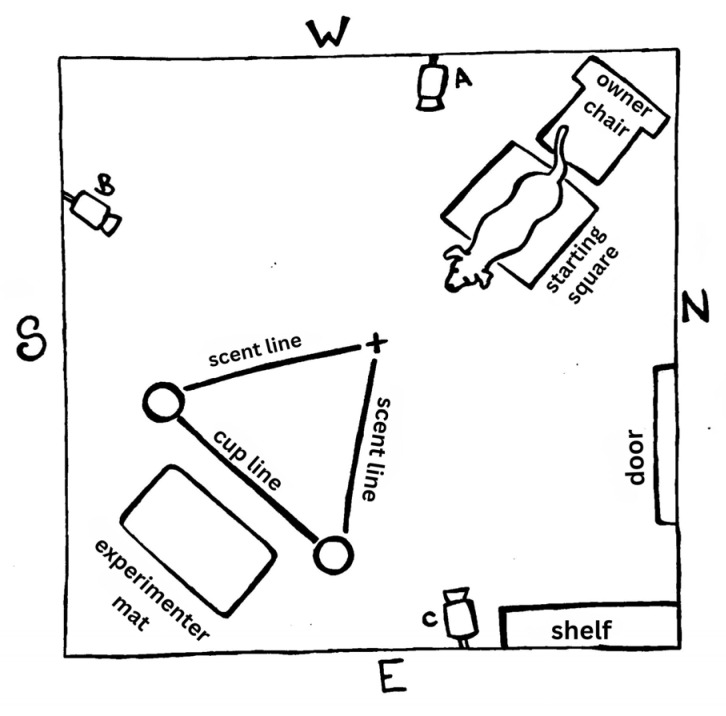
Example layout of the testing room showing owner chair position, subject starting box, cup line, scent lines, and experimenter mat position. There are three cameras labeled A, B, and C; cardinal directions are noted.

All trials were conducted in the Dog Cognition Lab at Barnard College in a 2.81 × 2.77 m testing room that was windowless and had a single door. There were three cameras (Lorex 1080p, Lorex, Markham, ON, Canada) positioned in the room to record the trials: two were mounted in the northeast and southwest corners of the room, and a third was placed on the floor on the southern side of the room. Additionally, there was a water bowl and a small cabinet in the southeastern corner. The owner was seated in a wooden chair in the northwestern corner and restrained the dog by the collar or a short leash between their legs to maintain the dog’s position within a taped floor square that marked the starting box (Figure 1).

The experimenter knelt on a 1.5 × 2.0 ft mat in the diagonally opposite corner. The floor in front of the experimenter’s mat was marked with orange tape to standardize the testing layout: a 3 ft horizontal line (“cup line”) directly in front of the experimenter’s mat with small circular markings indicating the left and right cup locations. The cups used were ceramic Solo cups. Two additional 3 ft tape lines (“scent lines”) extended symmetrically from the cup positions toward (but not reaching) the subject’s starting area, forming an equilateral triangle configuration which was used during olfactory pointing trials (Figure 1). During test trials, a 3-panel barrier (1.5 × 4 ft) was used by the experimenter to block the subjects’ view during cup-baiting. The floor was cleaned with a solution of 70% isopropyl alcohol between subjects to reduce any residual scent cues that could impact the dog’s odor-based decision-making [1]. The temperature and humidity of the room were recorded at the beginning of the trials. The temperature ranged from 70.7 to 76.8 degrees F (mean = 72.87 degrees F), and the humidity ranged from 18% to 27% (mean = 21.83%).

### 2.4. Trials

In both Phase A and Phase B, each subject completed warm-up trials and two types of test trials: four visual pointing trials and eight olfactory pointing trials. The olfactory pointing trials were evenly divided between two odor conditions: four “treat odor” trials and four “owner odor” trials. Throughout testing, experimenters communicated with the dog using dog-directed speech (e.g., “Hi Puppy, what’s this?”) to maintain subject attention during stimulus presentation [32]. To attempt to control for potential olfactory cues in the cups themselves, all cups were lightly rubbed with a treat (cheddar or salmon) along the interior bottom prior to use so that both baited and non-baited cups carried a similar treat odor. During Phase B, a few procedural variations were implemented with an interest in increasing robustness. Visual pointing trials were run prior to olfactory pointing trials, and baiting order was alternated systematically (RLRL or LRLR) to avoid contributing to any potential side bias. Owner-odor collection was modified (hand odor in Phase A; shoe odor in Phase B: see below) to explore alternative methods of capturing human scent, as there is limited precedent for standardized human odor collection in this type of paradigm. Cotton scent strings used in Phase A were replaced in Phase B with wider cotton strings (width = 0.5 in) to increase surface area and promote greater odor absorption and transfer. No other aspects of the experimental procedure were altered.

#### 2.4.1. Odor Collection and Olfactory Point Preparation

During olfactory pointing trials, cotton strings (3 ft long) were laid on the floor to provide odor cues from the stimulus (baited/non-baited cups) to the subject [29,30]. For each trial, one string contained a scent (treat odor or owner odor), and the other served as a control and was not exposed to any added odorant. Both strings were attached to the stimulus cups in the same fashion and were visually identical. The experimenter wore latex gloves during odor preparation to prevent odor contamination and changed gloves between preparations of odorized and control strings. During trials, strings were looped around themselves and stored within the cup they were taped to until use.

#### 2.4.2. Treat Odor Collection

For treat-odor trials, the cotton string was placed inside a sealed bag of dog treats (salmon or cheddar flavor) and shaken for 20 s. The string was then removed, and one end was taped to a pre-labeled ceramic Solo cup. 

#### 2.4.3. Owner Odor Collection

Owner odor was collected from participants’ hands during Phase A and from participants’ shoes during Phase B. For hand scent collection, an experimenter accompanied the owner as they washed their hands with water only and dried them thoroughly with a paper towel. The owner was then asked to roll the cotton string between their palms for 20 s using a rubbing motion [33]. The experimenter, wearing latex gloves, then placed the string in a glass bowl and returned it to the lab. For foot scent collection, the owner removed one shoe, and the experimenter, wearing latex gloves, rubbed the cotton string along the interior surface of the shoe for 20 s. In both cases, one end of the scented string was then taped to a pre-labeled ceramic Solo cup. Unscented control strings were handled with gloved hands.

### 2.5. Experimental Procedure

An experimenter met each owner-dog pair at a street-level entrance to Barnard College and escorted them to the testing room. Upon arrival, subjects’ leashes were removed, and they were allowed to explore the room freely for approximately 5 min to acclimate to the space. During this period, an experimenter (E1) explained the owner’s role in the study, and a second experimenter (E2) collected the owner’s odor sample (hand or shoe, depending on phase). Once subjects were judged to have reached a calm state, based on behavioral indicators such as reduced activity, moving away from the owner, and relaxed postures (e.g., lying down) [34], the owner sat in a designated chair and positioned the subject inside the taped starting square. Owners wore dark sunglasses during test trials to minimize inadvertent visual cueing. To remain as neutral as possible, experimenters avoided talking to, staring at, or petting the subjects until after trials were concluded. 

#### 2.5.1. Warm-Up Trials

Warm-up trials were conducted to strengthen the association between correct cup choice and reward. The warm-up protocol was based on the ManyDogs et al. paper [22], using fewer trials (3–10 warm-up trials rather than the ManyDogs Project’s 10–20 warm-up trials). The dog was positioned in the start box. The experimenter (E1) held up a treat at chest height, made eye contact with the subject, and said, “Hi puppy, what’s this?” The treat was then placed visibly at the center of the cup line. E1 sat back on her heels, looked down toward her lap, and said, “Go” to cue the release of the subject. After the subject consumed the treat, E1 said, “That’s it!” and the owner brought the subject back to the starting position. 

Next, a single cup was placed at the center of the cup line. E1 again held up a treat, made eye contact with the dog, and said, “Hi puppy, what’s this?” E1 visibly lifted the cup, placed the treat underneath, and replaced the cup. She then sat back on her heels, resumed a neutral posture, and said, “Go.” When the subject touched the cup or brought their nose within 5 cm of it, the cup was lifted, and the subject was permitted to consume the treat. To generalize the association across spatial locations, this procedure was repeated with the cup placed in the left and right positions of the cup line. Before each placement, E1 again held up the treat and said, “What’s this?” At the conclusion of warm-up trials, E1 said, “That’s it!” and the owner retrieved their dog. If E1 judged that the dog did not appear to understand the warm-up trials, she repeated them at her discretion.

#### 2.5.2. Test Trials: Visual Point

There were four pointing trials. Baiting location alternated between right (R) and left (L) across trials and was counterbalanced as to the initial side. The subject was positioned in the start box. E1 placed two cups on the cup line and hid them from view behind the barrier. E1 held up a treat and made eye contact with the subject, saying, “Hi puppy, what’s this?”, then placed the treat under either the R or L cup according to predetermined order. The barrier was removed, and the cups were slid out to their marked right and left positions. E1 sat back on her heels and pointed to the baited cup with her contralateral hand (cross-body point), letting her gaze follow her hand, and said, “What’s this?” once. This pointing position was held for approximately 2 s. E1 then withdrew her arm, placed both hands behind her back, directed her gaze downward, and said, “Go.” While E1 counted in her head to 30 s, the subject was released. Subjects were given 30 s to make a choice.

If the subject touched a cup with snout, mouth, or front paw, or brought their nose within 5 cm of a cup, the experimenter marked this as a selection. If the subject selected the baited (correct) cup, the cup was lifted, and the subject was allowed to consume the treat. If the subject selected the unbaited (incorrect) cup, that cup was lifted, and the baited cup and treat were immediately retrieved before the subject could access them. No reward was given on incorrect trials. If the subject did not start moving in the first few seconds, E1 said “again!” and asked the owner to release their dog with an “okay” or “go get it!” The trial was ended if the subject did not approach either cup in 30 s; this was recorded as a “no choice.”

#### 2.5.3. Test Trials: Olfactory Point

Each subject was presented with eight olfactory pointing trials. The general setup, cup placement, timing, release procedure, and choice criteria were identical to those of the visual pointing trials, except as specified below. In each trial, a treat was hidden under one of two cups, and olfactory points were presented using scent-trails (cotton strings) that provided odor cues to the cup locations. As in the visual pointing trials, two cups were placed on the cup line and occluded by a barrier during baiting. At the start of each trial, E1 attracted the subject’s attention by saying, “Hi puppy, what’s this?” while visibly showing the treat. The treat was placed under the designated cup. The baited cup was attached to either a treat-odor string or an owner-odor string; the alternative cup was attached to a visually identical string without added odor, serving as a control.

The barrier was removed and placed behind E1, and the cups were slid into their marked left and right positions. E1 then pulled both strings forward simultaneously along the scent lines. As the strings reached full extension, E1 said, “What’s this?!” and tapped her fingers on the ground at the ends of the strings to draw attention to the scent trails.

**Figure 2:**
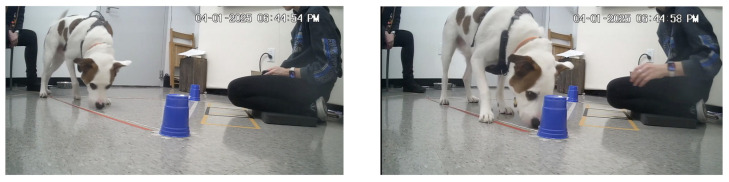
Examples of subject (Beto) making a choice of a cup during olfactory point trials.

Subjects were again given 30 s to make their choice, and the choice was coded using the same criteria as in the pointing trials (Figure 2). Reward and incorrect-trial procedures were also identical to those described above. A trial was recorded as a “no choice” if the subject did not approach either cup within 30 s. In all trials, if a subject approached a string but did not proceed toward the corresponding cup, the experimenter prompted a re-release by saying, “Again.” If a subject attempted to ingest (rather than lick) a string, the string and associated cup were immediately removed. Trials were randomized. Individual strings were reused across trials within a single dog’s session and were replaced for each subject.

### 2.6. Behavioral Coding

All subjects were recorded by video cameras during trials for later frame-by-frame playback (30 fps). For each trial, both trial completion (i.e., whether the subject made an approach to a cup) and trial outcome (correct vs. incorrect) were recorded. Subjects’ latency to approach was recorded for each trial; however, this measure was used only to confirm whether a choice occurred within the 30 s response window. Choice was defined as the subject touching a cup with the snout, mouth, or front paw, or bringing the snout within 5 cm of a cup. Behavioral videos were coded independently by two of the lab experimenters. Coders were blind to the baiting side (right vs. left) in all trials and to odor condition (owner-odor vs. treat-odor) in olfactory pointing trials. Inter-rater reliability was assessed on 40% of trials by two independent coders and calculated as percent agreement (i.e., the proportion of trials on which both coders assigned the same outcome: successful vs. unsuccessful). Agreement exceeded 99%. Data was analyzed in R (Version 2025.05.0+496) to assess for significant successful results above chance, as well as comparisons across conditions and between Phases A and B. 

## 3. Results

Forty-eight dogs participated in the pointing trials for this study. A binomial test was conducted on aggregated trials for Phases A and B to determine whether subjects selected the correct cup at a rate above chance (0.5).

### 3.1. Subject Behavior on Visual Pointing Trials

**Figure 3:**
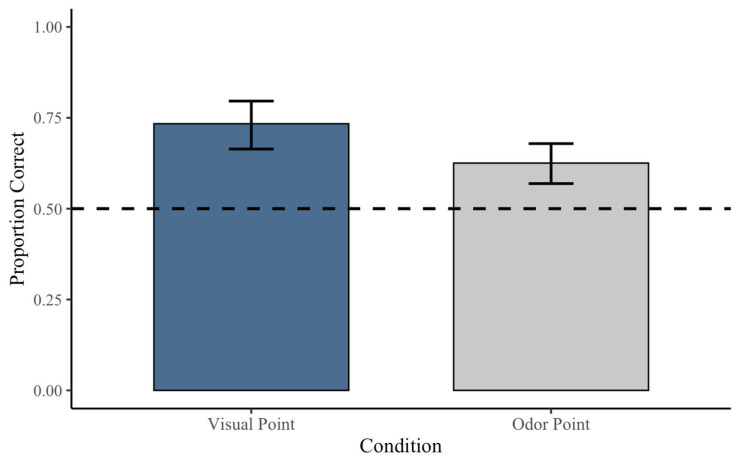
Proportion of correct choices by subjects across visual pointing trials and combined olfactory trials. Subjects selected the correct cup at rates significantly above chance (0.5) in both conditions, with higher performance in visual pointing trials (73.4%) than olfactory trials (62.5%). Error bars represent 95% confidence intervals.

Across 184 pointing trials, subjects selected the correct cup on 135 trials (73.4%). Performance was significantly above chance, *p* < 0.001, 95% CI [0.664, 0.796]. The effect size was medium-to-large (Cohen’s *h* = 0.49), indicating a substantial difference from chance performance (Figure 3).

### 3.2. Subject Behavior on Olfactory Pointing Trials

Not all subjects completed every olfactory pointing condition; therefore, sample sizes vary across odor analyses. Across all olfactory point trials combined (treat and owner odor), subjects selected the correct container on 197 of 315 trials (62.5%). Performance was significantly above chance, *p* < 0.001, 95% CI [0.569, 0.679], with a small-to-medium effect size (Cohen’s *h* = 0.25) (Figure 3).

#### Treat and Owner Odor Trials

**Figure 4:**
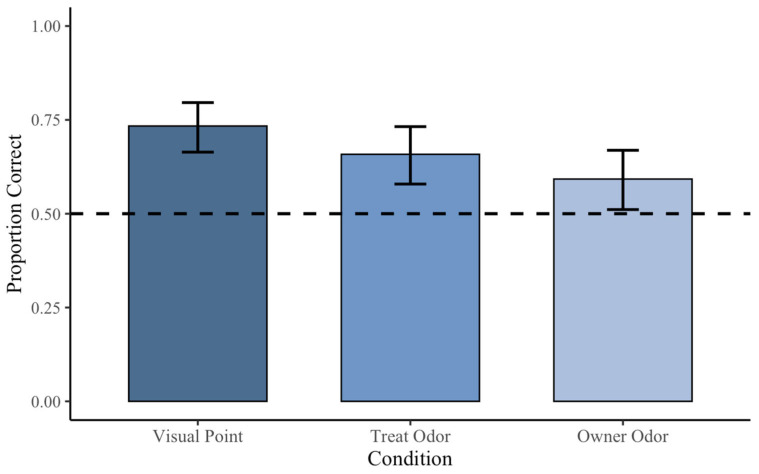
Proportion of correct choices by subjects in olfactory trials separated by odor type. Subjects performed above chance in both treat odor (65.8%) and owner odor (59.2%) conditions, with slightly higher accuracy in treat odor trials. Error bars represent 95% confidence intervals.

Each kind of odor trial was analyzed separately as well. For treat odor trials (*n* = 44 dogs), subjects chose the correct container on 104 of 158 trials (65.8%). Performance was significantly above chance, *p* < 0.001, 95% CI [0.579, 0.732], with a small-to-medium effect size (Cohen’s *h* = 0.32). For owner odor trials (*n* = 42 dogs), subjects selected the correct container on 93 of 157 trials (59.2%). Performance was significantly above chance, *p* = 0.013, 95% CI [0.511, 0.669], with a small effect size (Cohen’s *h* = 0.19) (Figure 4).

### 3.3. Individual Performance Across Trials

**Figure 5:**
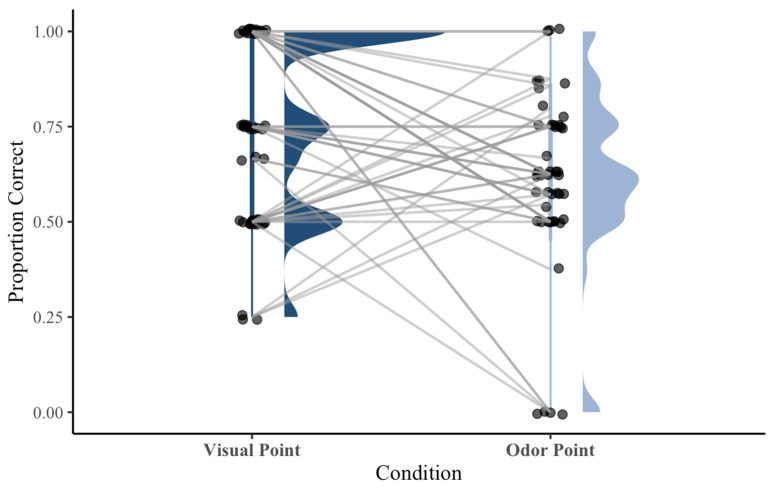
Individual subject performance in visual point and olfactory point conditions. Each point represents a subject, with gray lines indicating paired performance across conditions. Raincloud plots display data distributions (kernel density) and median with 95% confidence intervals. Subjects performed significantly better on visual pointing trials than on olfactory-pointing trials (*p* = 0.02).

To directly compare performance between treat and owner odor conditions, a Wilcoxon signed-rank test was conducted at the subject level. There was no significant difference in performance between subject performance on treat odor trials (Mdn = 0.71, IQR = 0.25) and owner odor trials (Mdn = 0.50, IQR = 0.25), *Z* = −1.05, *p* = 0.294, *r* = 0.16, indicating a small effect size. To compare overall performance across subjects between visual pointing and total olfactory pointing trials, a Wilcoxon signed-rank test was conducted. Subjects performed significantly better on pointing trials (Mdn = 0.75, IQR = 0.50) than on scent trials (Mdn = 0.625, IQR = 0.25), *Z* = −2.34, *p* = 0.019, *r* = 0.34, with a power of 0.80 (n = 47, Cohen’s *d* = 0.42) (Figure 5).

### 3.4. Performance Between Phases

**Figure 6:**
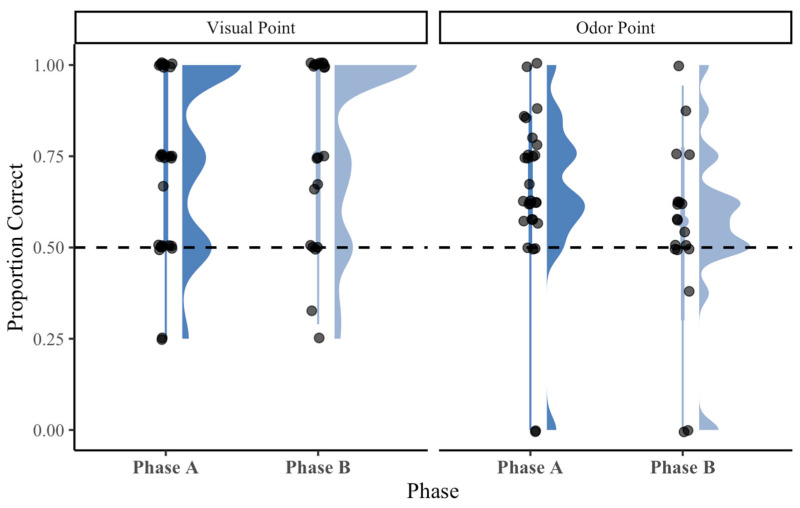
Subject performance across Phase A and Phase B for visual point and olfactory point conditions (faceted by condition). Raincloud plots display distributions and individual observations. The dashed line indicates chance performance (0.5). No significant phase differences were observed.

To assess whether performance differed between study phases, a Mann–Whitney U test was conducted on Phase A vs. Phase B visual pointing trials and on Phase A vs. Phase B olfactory pointing trials. There was no indication of a significant difference between Phase A and Phase B visual pointing performance, *p* = 0.519. There was no indication of a significant difference between Phase A and Phase B olfactory pointing performance, *p* = 0.055, *Z* = −1.91, *r* = 0.28. These results suggest that subjects’ performance did not differ meaningfully between phases for either visual or olfactory pointing trials (Figure 6). 

## 4. Discussion

This research introduces a novel way of approaching the classic object-choice task (OCT): Creating an olfactory analog to the pointing gesture. Using a method of pointing based on smell in addition to a visual point allows us to further investigate the role of odor cues in dogs’ decision-making and how they respond to different communicative modalities. Our goal was to explore dogs’ cognitive abilities within a framework that reflects their sensory world. As expected, during visual pointing trials, subjects correctly followed the experimenter’s ostensive point at a rate significantly higher than chance [10,11,12,21]. The most notable finding of the present study occurred during olfactory-point trials, where subjects followed the odor “point” to the correct cup at rates significantly higher than chance as well. We found no significant difference in performance between trials using treat-odor-scented strings and those using owner-odor-scented strings. Together, these findings demonstrate that dogs can use experimentally presented olfactory cues to guide choice-making behavior within an object choice framework. This behavior occurred even when a human experimenter was present and had previously communicated using visual gestures. These findings suggest that odorants in testing environments may act as meaningful sources of information for the dog [1].

Many object-choice studies include ostensible “odor controls” to determine whether dogs rely on smell rather than the human pointing gestures to locate the baited container. Often in these control trials, one cup remains baited, but the visual point is removed [22,24,25,35]. If dogs fail to succeed above chance in these smell-only trials, then it is often used as evidence that dogs do not rely on olfaction when solving a task [22]. Our findings suggest that this interpretation may be incomplete. Rather than demonstrating a preference for visual information, these findings likely reflect earlier experience and conditioned expectations that the dog may hold. Because domestic dogs are so highly familiarized with human gestural communication [36,37], they may interpret the removal of a pointing cue as the absence of guidance rather than as an introduction of the opportunity for a separate method of communication. In this sense, previous smell-only trials may test dogs’ capability to reinterpret the task, rather than their ability to use odor cues as information. The present study reimagines these smell-only trials. We preserve the gesture and communicative structure of the point, but we translate it into an odorant point as a complement to the earlier visual one.

An examination of the data reported [1,2] by the ManyDogs Project [22] further supports this interpretation. In their study, dogs performed slightly better in the ostensive pointing condition (M = 0.53) than in the odor-control condition (M = 0.51): Statistically varying from chance in the pointing condition but not in the odor-control condition. However, no analysis was made between the two conditions. An approximate comparison of the reported summary statistics suggests that this difference was not statistically significant (t ≈ 1.56, *p* ≈ 0.12). This pattern indicates that dogs’ performance in smell-only trials may not differ meaningfully from their performance when following gestures, despite the common interpretation that such trials demonstrate a reliance on vision. Taken together with the present findings, there is good reason to believe that dogs are capable of using olfactory information when it is structured clearly and intentionally within the task, rather than requiring them to spontaneously switch their sensory approach.

In the present study, odor was made spatially informative, improving upon the simple “smell-at-the-cup” detection model used in many odor control paradigms [22,24,25]. Just as dogs can follow the direction of an outstretched finger, they may also follow the implied direction of a scent-trail laid by an experimenter in front of them. This use of odor as a communicative signal is an important methodological contribution to the field of canine cognition. 

In considering methodological details, a few points are of note. First, analysis of data collected across two phases (A and B) revealed no significant differences in performance between Phase A and Phase B, suggesting that the present findings are robust across these variations in design.

Second, within the olfactory-point condition, dogs did not follow the treat-odor trail at a significantly higher rate than the owner-scented trail. Intuitively, one might expect that a strong food or treat smell would be more salient than a human smell; owners were even asked to bring their dogs’ favorite treats to maximize food motivation. However, in this study, both comestible and social cues were informative and supported above-chance performance. Notably, most canine cognition research findings note food odors in the testing environment but do not make note of any odor cues that may inadvertently be left by people and other dogs who have been in the environment. This occurs even though dogs are known to differentiate individual humans by odor and represent familiar people through olfaction [2]. 

Third, subject performance on the visual pointing task was substantially higher (73%) than that reported in some previous OCT studies, including the ManyDogs Project [22]. Several methodological differences may account for this pattern. Our study included fewer subjects (N = 48) and only four pointing trials per dog, which may increase variability in performance. Additionally, the pointing gesture used in our experiment was highly ostensive: the experimenter used a clear cross-body point accompanied by obvious gaze direction and dog-directed speech (“What’s this?”). The explicit and communicative nature of this gesture may have been particularly salient for the subjects [18,19,31,38]. 

The present study also contributes to a growing effort within comparative cognition to redesign experimental paradigms in ways that better align with the sensory capacities of nonhuman animals [6,7,8]. Such work demonstrates how established cognitive paradigms can be reconfigured to better match the dominant sensory modalities of different species. The present study extends this approach by adapting the communicative structure of the object-choice task into an olfactory modality.

Overall, the present findings suggest that dogs are capable of using olfactory information to guide decision-making when that information is structured clearly within a communicative framework. Rather than treating odor as a confounding variable to simply be controlled, these results support the view that olfaction can serve as a meaningful and interpretable signal for dogs performing cognitive tasks. Future research should continue to explore how different sensory modalities can be integrated into experimental design, including testing whether dogs can learn to flexibly switch between visual and olfactory cues within the same task. Designing paradigms that better reflect the sensory world of our subject animal may ultimately lead to a more accurate understanding of how nonhuman species perceive, interpret, and solve problems.

## 5. Conclusions

Subject dogs demonstrated the ability to use both visual and olfactory cues to guide decision-making within an object-choice framework. When odor was structured as a communicative, spatially informative signal, dogs followed these “olfactory points” at rates above chance, though performance remained stronger for highly visible intentional pointing and gaze. These findings suggest that olfaction can function as a meaningful communicative modality for dogs. Adapting classic paradigms to better reflect species-specific sensory systems may provide a more accurate understanding of nonhuman cognition.

## Figures and Tables

**Table 1:** Information on subjects who participated in the study during Phase A and Phase B: their name, age (yrs), sex, and breed (as reported by the owner).

**Phase A Subjects**			
**Name**	**Age in Years (Rounded)**	**Sex**	**Breed** **(as Reported by Owner)**
Magnus	5	Male	Springer Spaniel
Matisse	4	Male	West Highland Terrier
Riley	4	Male	Australian Silky Carrier
Kipper	4	Male	Lagotto Romagnolo
Bella	10	Female	Toy Poodle
Poppy	4	Female	Poodle, Golden Retriever
Bubbles	3	Male	Shih Tzu—Poodle
Dana	5	Female	American Bully, American Pit Bull Terrier, Beagle, Chow Chow, and more
Rexie	5	Male	Maltese—Shih Tzu
Hermes	10	Male	Yorkshire Terrier
Penny	12	Female	Corgi, Dachshund, Border Collie
Kloppi	2	Female	Jack Russell/Rat Terrier/other mix
Tasha	12	Female	Havanese
Nugget maltet	5	Female	Cockapoo
Pedro	7	Male	Sato (Puerto Rican mutt)
Citi	9	Female	American Pitbull Terrier
Petey	8	Male	Chihuahua, Miniature Pinscher
Ozzy	4	Male	Staffy, German Shepherd, Husky, Great Pyrenees, Malamute
Zen	8	Female	Golden Retriever
Charley	13	Female	Miniature Poodle
Phoebe	3	Female	Plot hound
Rusty	13	Female	Shih Tzu and Bichon Frise
Beto	5	Male	Mixed Breed
Nani	11	Female	Shiba Inu
Theo	3	Male	Australian Shepherd
Hans	5	Male	APBT/Mountain Cur/GSD/Supermutt
Rooney	8	Male	Bassett Hound, American Staffordshire Terrier
Cady	8	Female	American Cattle Dog Mix
**Phase B Subjects**			
**Name**	**Age in Years (Rounded)**	**Sex**	**Breed** **(as Reported by Owner)**
Nessa	7	Female	Old English Sheepdog + Poodle
Jotunn	2	Male	Border Collie Sheepdog and Mix
Toby	11	Male	Mixed Breed
Taquita Louise Bell	3	Female	Chihuahua
Ladybird	4	Female	Pitbull, Mountain Cur, Yellow Lab, German Shepherd, Chow Chow
Zishunka	5	Female	Australian Cattle Dog, Golden Retriever, Stafford Terrier
Margaret	1	Female	Bernese Mountain Dog and Poodle
Agnes	4	Female	Beagle
Luca	2	Male	Mixed Breed
Nugget	1	Female	Mini Goldendoodle
Ginger	5	Female	Mini Aussie
Nico	2	Male	American pitbull and lots of other stuff
Owen	5	Male	Welsh springer spaniel
Major	1	Male	Poodle and Newfoundland
Felix	8	Male	Border Terrier/Jack Russell Mix
Suede	4	Female	Labrador Retriever, American Pitbull Terrier, Beagle, Doberman, Mutt Mix
Pidge	9	Female	German Wirehaired Pointer
Argent	1	Female	Shetland Sheepdog
Mesa	2	Female	American Pitbull Terrier, Labrador Retriever, American Foxhound, Chow Chow
Clover	4	Female	Australian Shepard and Poodle mix
Pippa	9	Female	Sealyham Terrier

## Data Availability

The data referenced in this manuscript will be made available by the authors, without undue reservation, to any qualified researcher upon request.

## Abbreviations

The following abbreviations are used in this manuscript:

OCT	Object-choice task
